# Temperature and Food Influence Shell Growth and Mantle Gene Expression of Shell Matrix Proteins in the Pearl Oyster *Pinctada margaritifera*

**DOI:** 10.1371/journal.pone.0103944

**Published:** 2014-08-14

**Authors:** Caroline Joubert, Clémentine Linard, Gilles Le Moullac, Claude Soyez, Denis Saulnier, Vaihiti Teaniniuraitemoana, Chin Long Ky, Yannick Gueguen

**Affiliations:** 1 Ifremer, UMR 241 « Ecosystèmes Insulaires Océaniens », Labex Corail, Centre du Pacifique, Taravao, Tahiti, Polynésie Française; 2 University of French Polynesia, UMR 241 « Ecosystèmes Insulaires Océaniens », Faa'a, Tahiti, Polynésie Française; Faculdade de Medicina Dentária, Universidade do Porto, Portugal

## Abstract

In this study, we analyzed the combined effect of microalgal concentration and temperature on the shell growth of the bivalve *Pinctada margaritifera* and the molecular mechanisms underlying this biomineralization process. Shell growth was measured after two months of rearing in experimental conditions, using calcein staining of the calcified structures. Molecular mechanisms were studied though the expression of 11 genes encoding proteins implicated in the biomineralization process, which was assessed in the mantle. We showed that shell growth is influenced by both microalgal concentration and temperature, and that these environmental factors also regulate the expression of most of the genes studied. Gene expression measurement of shell matrix protein thereby appears to be an appropriate indicator for the evaluation of the biomineralization activity in the pearl oyster *P. margaritifera* under varying environmental conditions. This study provides valuable information on the molecular mechanisms of mollusk shell growth and its environmental control.

## Introduction

The development of marine tropical mollusks through their life cycle depends on variations in water temperature and food availability. Many studies have shown that food and temperature play an essential role in somatic and shell growth [1]–[4]. In particular, it was seen that the growth of each species has a particular optimal thermal environment [5]–[8]. The trophic environment has been shown to act on growth even if the environment is considered as oligotrophic [9]. For example, in French Polynesia, Pouvreau and Prasil [10] observed differential *P. margaritifera* shell growth between different types of island (atolls *vs* high islands), where nutritional and temperature conditions are different. Furthermore, Kvingedal *et al.* [11] demonstrated the importance of phytoplanktonic diversity on *P. maxima* spat growth and Linard *et al.* [12] showed that shell growth of *Pinctada margaritifera* depended on food concentration.

Shell secretion by mollusks is a matrix-mediated biologically-controlled biomineralization process that takes place outside the living tissues [13]. Mollusk shell is a natural biomaterial made up of a mineral phase - calcium carbonate (CaCO_3_) - and an organic cell-free matrix secreted by the external mantle epithelium, the tissue layer underlying the shell. This extracellular calcifying organic matrix is a minor constituent of the shell [14], and is mainly composed of proteins [15]. This calcifying shell matrix interacts with the crystal surface to orientate its nucleation and control CaCO_3_ crystal polymorphisms, in the form of aragonite or calcite [15]–[16], leading to the laying down of different microstructural layers of the shell. Recently, the number of genes identified as coding for molluskan shell matrix components has increased [17]–[20], revealing the wide variety of proteins implicated in the biomineralization process [21]–[23]. Numerous matrix proteins from *P. margaritifera* shell have also recently been identified from the nacreous and prismatic layers [15]. These authors provided strong evidence showing that the proteinaceous matrices associated with prism and nacre are extremely different.

In this study, we aim to analyze the combined effect of environmental conditions, microalgal concentration (800 and 15000 cell mL^−1^) and temperature (21, 25 and 28°C), on shell growth and the molecular mechanisms underlying this biomineralization process in the bivalve *P. margaritifera*. Shell growth was measured using calcein staining of calcified structures [12]. Molecular mechanisms were studied by following the level of expression of a set of 11 genes implicated in the biomineralization process in the mantle, the calcifying tissue of the shell [17]. We showed that shell growth is influenced both by microalgal concentration and temperature and that these environmental factors also regulated the expression of most of the genes targeted in this study.

## Materials and Methods

### Biological material

Cultivated black-lip pearl oysters *P. margaritifera* of a mean height of 85±6 mm and mean weight of 80±14 g (n = 60) were obtained from the Ifremer Center at Vairao lagoon, Tahiti, French Polynesia.

### Calcein shell marking

Calcein was purchased from Sigma Aldrich, France. The stain powder was dissolved over 12 h at 24°C in filtered seawater (0.1 µm) using a magnetic stirrer. Pearl oyster shells were marked by immersion of pearl oysters in 150 mg L^−1^ calcein solution for 12 h [12].

### Experimental design

Sixty pearl oysters placed in 6 500-L tanks were cultured for 60 days. For each tank, seawater was renewed at the rate of 100 L.h^−1^ (20% per hour). Three temperatures (21°C, 25°C and 28°C) and two microalgal concentrations, 800 cell mL^−1^ for low food (LF) and 15 000 cell mL^−1^ for high food (HF), were tested. The pearl oysters were fed with a mixed (v:v) diet composed of the microalgae *Isochrysis galbana* (T-Iso) and *Chaetoceros gracilis*, supplied continuously with an Ismatech pump. Six combinations (temperature/food level) were tested to provide a range of environmental conditions: 21/LF, 21/HF, 25/LF, 25/HF, 28/LF and 28/HF. Ten pearl oysters were placed in each environmental combination.

Such an experimental protocol, imposed by the different experimental conditions tested, could induce a pseudo-replication issue. However, based on our experience in this experimental structure on similar topics, this experimental design has already demonstrated its effectiveness [12].

### Sampling

After 60 days, all the pearl oysters were dissected to collect a piece of mantle. The mantle of 10 pearl oysters, per condition, was sampled. Realizing the practical limitations imposed by the number of samples, conditions and genes tested, we decided to group the samples into 2 pools. Each pool comprised the pieces of mantle of 5 pearl oysters randomly chosen among the 10 per condition. Such a pooling sampling strategy was already demonstrated to be effective in identifying the most common changes in gene expression profile even if the variability in gene expression from one individual tissue sample may obscure common patterns of gene expression [24]. The shells were kept to be analyzed individually.

### Shell deposit rate measurement

**Figure 1:**
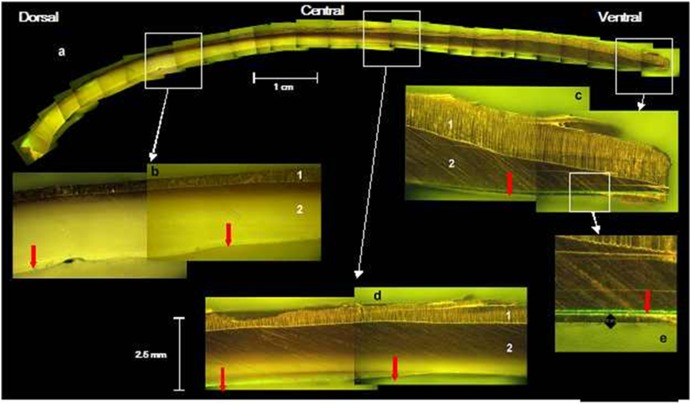
Section of a valve of a *P. margaritifera* shell. (a) dorso-ventral side showing the transition areas between nacreous and prismatic layers. (b) dorsal part; (c) ventral part; (d) central part; (e) detail of ventral part. (1) prismatic calcite; (2) aragonite tablets. The red arrows indicate the calcein mark.

To investigate shell growth, the shells were sawn using a “Swap Top Trim Saw” machine (Inland, Middlesex, United Kingdom), which includes a diamond Trim Saw Blade (Thin Cut) IC-40961. Shell edges were then polished for 5 sec with various grades of water sand paper sheet. The shell sections were then examined under a Leitz Dialux 22 compound fluorescence microscope equipped with an I3 filter block and an optical micrometer. Shell growth was measured by evaluating the thickness of deposits at the ventral side of the shell, based on the calcein marks, using the optical micrometer [12]. Shell deposit rate (SDR) was calculated by dividing the thickness of deposits by the time elapsed since the marking. SDR is expressed in µm.d^−1^. The aragonite tablet, the prismatic calcite and the transition areas between nacreous and prismatic layers of the shell are visible using optical micrometer (Figure 1). During shells sawing, ten of them were broken and became unusable.

### Mantle gene expression

The two pools of calcifying mantle samples were analyzed for each of the six sets of environmental conditions studied (five individuals/pool). Total cellular RNA was extracted for each of the 12 pools using TRIZOL Reagent (Life Technologies) according to manufacturer's recommendations. RNA integrity was assessed using 1% agarose gel analysis. RNA was quantified using a NanoDrop ND-1000 spectrophotometer (NanoDrop Technologies Inc). The expression levels of 11 genes were analyzed by quantitative RT-PCR analysis using a set of forward and reverse primers (table 1). A universal set of primers for the 18S rRNA gene sequence was used as a first reference gene; these had first been designed based on the alignment of different bivalve species (Uni1304FTTAGTTGGTGGAGCGATTT/Uni1670R TAGCGACGGGCGGTGTG) [25]. A second reference gene was used and chosen based on its ubiquitous and constitutive expression pattern in *P. margaritifera* tissue (REF1S AGCCTAGTGTGGGGGTTGG/REF1AS ACAGCGATGTACCCATTTCC). First strand cDNA was synthesized from 400 ng total RNA using Transcriptor First Strand cDNA Synthesis Kit (Roche) and a mix of poly(dT) and random hexamer primers. qPCR amplifications were carried out on a Stratagene MX3000P, using Brilliant II SYBR Green QPCR Master Mix (Stratagene) with 400 nM of each primer and 1 µL of cDNA template. The following run protocol was used: initial denaturation at 95°C for 10 min followed by 40 cycles of denaturation at 95°C for 30 s, annealing at 60°C for 1 min and extension at 72°C for 30 s. Lastly, the amplicon melting temperature curve was analyzed using a melting curve program: 45–95°C with a heating rate of 0.1°C×s-1 and a continuous fluorescence measurement). All measurements were made in duplicate. The comparative Ct (threshold cycle) method was used to analyze the expression levels of the genes of interest. All analyses were based on the Ct values of the PCR products. The relative expression ratio of each analyzed length of cDNA was calculated based on the delta–delta method normalized with two reference genes for comparing the relative expression results, which is defined as: ratio = 2^−[ΔCt sample–ΔCt calibrat^°^r]^ = 2^−ΔΔCt^ [26]. Here the ΔCt calibrator represents the mean of the ΔCt values obtained for all tested genes.

**Table 1:** Set of forward and reverse primers used for the gene expression analysis.

Gene	GenBank Accession Number	Forward primer	Reverse primer
PIF 177	BAH97338	5′-AGATTGAGGGCATAGCATGG-3′	5′-TGAGGCCGACTTTCTTGG-3′
MSI60	BAA20466	5′-TCAAGAGCAATGGTGCTAGG-3′	5′-GCAGAGCCCTTCAATAGACC-3′
Pearlin	ABG24165	5′-TACCGGCTGTGTTGCTACTG-3′	5′-CACAGGGTGTAATATCTGGAACC-3′
Linkine	ABO87300	5′-TTGTGGAAGTCAAGTCGTCAG-3′	5′-GCAGTAGTAGGCGTCCATCC-3′
Nacrein	BAA90540	5′-CTCCATGCACAGACATGACC-3′	5′-GCCAGTAATACGGACCTTGG-3′
Shematrin 8	ABO92760	5′-TGGAGGTGGAGGTATCGTTC-3′	5′-ACACCTGATACCCTGCTTGG-3′
Shematrin 9	ABO92761	5′-TGGTGGCGTAAGTACAGGTG-3′	5′-GGAAACTAAGGCACGTCCAC-3′
MPN88	BAH05008	5′-CTGGTCAACAAACAGGAGCA-3′	5′-ACCTCCTTGGGCTCCTAGTC-3′
Mantle protein 10	AAZ22319	5′-GCCCGTCCACAGAACTAGAG-3′	5′-GATGAGGCACGTCTTTGACC-3′
KRMP7	ABP57445	5′-GCCTTCACCACAGAAGGAAG-3′	5′-GCCGAATTTCTTCAGACACC-3′
Fibronectin	CCE46158	5′-GCGTCAAGACCTTACCCAAA-3′	5′-TCCTGTGTGACCGTGATTGT-3′

### Statistics

Normality of data distribution and homogeneity of variance were tested for shell deposit rate (SDR) and gene expression data using Shapiro-Wilk test and F-test.

SDR data were normally distributed and variances were homogenous. Hence, effects of food level and temperature, and their interaction on SDR, were tested using a two-way ANOVA. Multiple Range Tests (Tukey honest significant differences, HSD) were used to determine which means were significantly different from others. In all cases, differences were considered significant at the level p<0.05.

As the assumptions of the parametric ANOVA were not met for gene expression data, we used the Scheirer-Ray–Hare (SHR) non-parametric ANOVA [27] to test the effects of food level and temperature. SHR is a method used for the analysis of ranked data arising factorial design. This procedure is an extension of the Kruskal-Wallis ranks test that allows the calculation of interaction effect. Pairwise comparisons within the 3 levels of temperature were carried out using Mann-Whitney U-tests. Differences were considered significant at the level p<0.05.

Correlations between gene expression data and SDR were tested using the critical value table for Spearman's rank correlation coefficient *rho* at the 5% alpha level. The gene expression values of each pool were associated to the corresponding SDR values of each of th five pearl oysters having contributed to each pool. So, the correlation, for each gene, was tested using the 12 values of relative gene expression of each pooled samples with the 50 individual SDR data, whatever the environmental conditions. The null hypothesis was then rejected when *rho*<0.28.

## Results

### Deposit dynamics differ across the shell

Figure 1 shows the shell deposit dynamics in three places on the shell of the pearl oyster *P. margaritifera*. Deposition rate differs depending on the shell part considered. In the central part, the calcein mark remained on the surface on the inside of the shell (Figure 1d) while on the dorsal and ventral sides, it became covered with aragonite (Figure 1b, c, e). Figure 1 also shows that the shell is mainly composed of aragonite on its dorsal (about 80% of the shell thickness, (Figure 1b) and central parts (70%, Figure 1a), while calcite represents more than 70% of the shell thickness on the ventral side (Figure 1c, e).

### Shell deposit rate (SDR) is influenced by temperature and food

**Figure 2:**
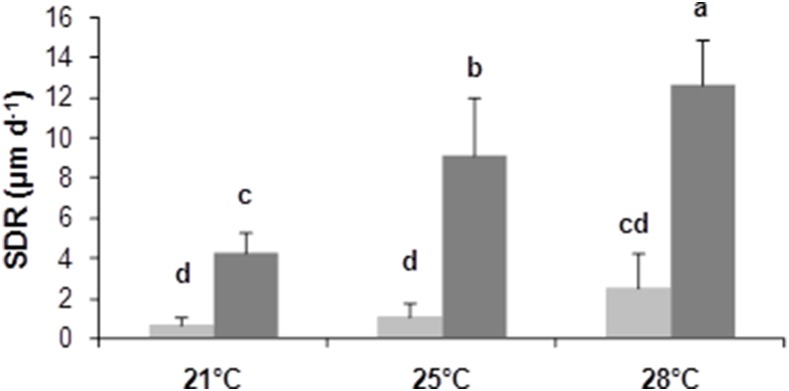
Shell deposit rate (µm.d^−1^) on the ventral side of the shell according to temperature and food concentrations over the two month experiment. Three temperatures (21, 25 and 28°C) and two microalgal concentrations (800 cell mL^−1^: light grey and 15000 cell mL^−1^: darkgrey) were tested. The five homogeneous groups identified by the Tukey post-hoc test (a, b, c and d) are indicated (means and standard error, n = 10).

SDR measurement was made using calcein marking in order to analyze the combined effect of environmental conditions (food and temperature) on shell growth. The two-way ANOVA showed a significant effect of temperature on SDR (F = 39.94, p<0.0001). The post-hoc Tukey test showed that SDR was significantly influenced by temperature with SDR_28_>SDR_25_>SDR_21_ (Figure 2). Food level also had a significant effect (F = 233.96, p<0.0001) and the Tukey test showed that SDR_HF_>SDR_LF_. A significant interaction between temperature and food level was detected (F = 16.59, p<0.0001).

### Mantle gene expression of shell matrix protein is influenced by temperature and food

The expression levels of 11 genes encoding proteins of the shell matrix were examined using quantitative Real-Time PCR (qRT-PCR) on *P. margaritifera* mantle samples. Among these genes, we selected four genes coding for proteins of the nacreous layer (*Pif 177, MSI60, pearlin, linkine,*), two coding for proteins identified in both nacreous and prismatic layers (*nacrein and shematrin 8*) and another five coding for proteins of the prismatic layer (*shematrin 9, MPN88, mantle protein 10, KRMP, fibronectin 1)* [15].

**Figure 3:**
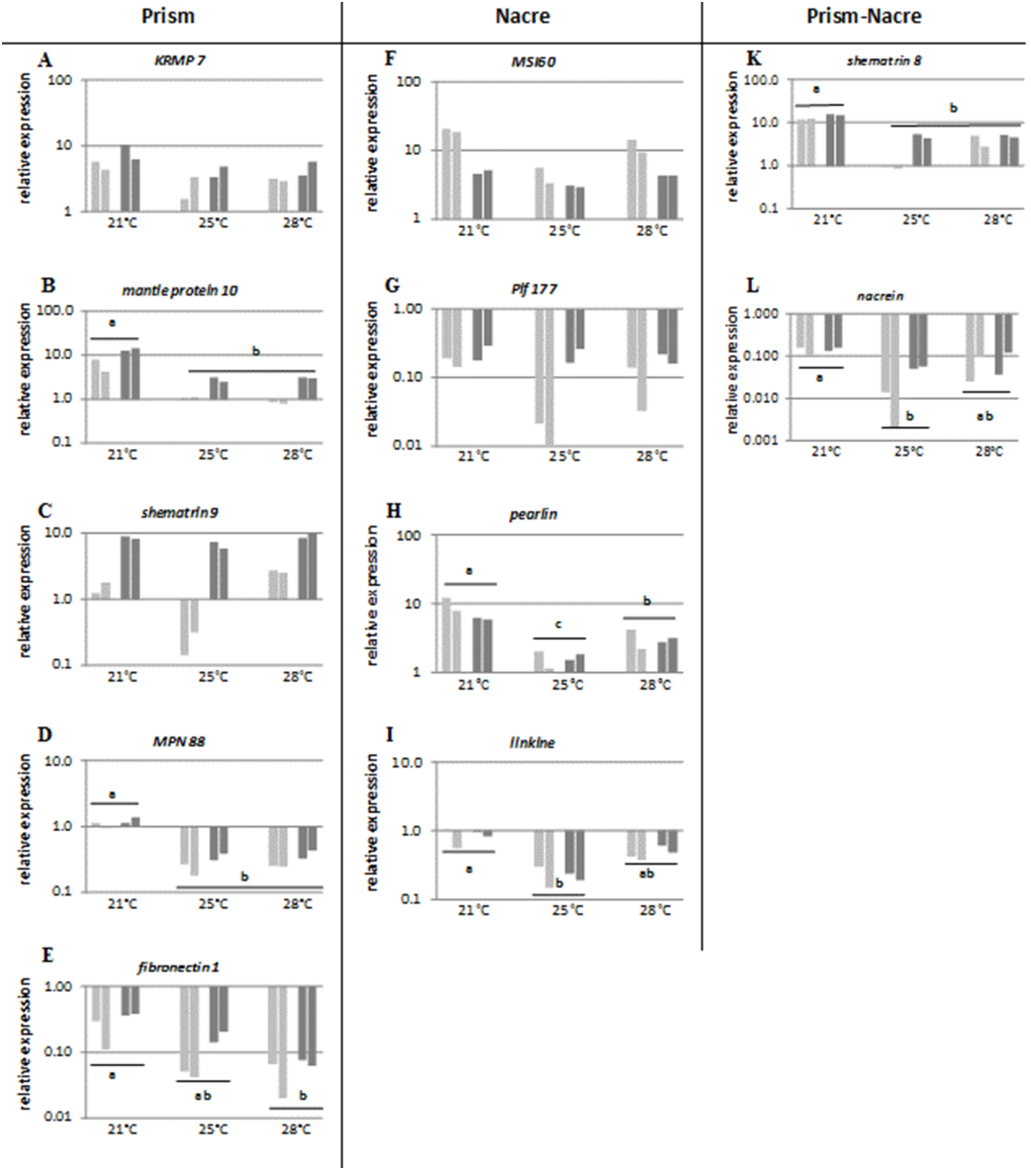
Relative gene expression of genes coding for proteins potentially involved in the construction of the prismatic layer (*KRMP 7, mantle protein 10, shematrin 9, MPN88, fibronectin 1)* the nacreous layer (*MSI60, Pif 177, pearlin, linkine)* and both the prismatic and the nacreous layers (*shematrin 8, nacrein)* following 2 months of exposure to 3 temperatures (21°C, 25°C and 28°C) and 2 microalgal concentrations 800 cell mL^−1^ for low food (LF) (light grey) and 15 000 cell mL^−1^ for high food (HF) (dark grey). The results of two measures performed on a pool of 5 pearl oysters are shown for each condition. Statistical differences between temperatures are indicated by a letter.

By comparing the level of expression of each of the target gene in defined environmental conditions with the expression levels obtained for all the 11 genes and six tested environmental conditions, our results showed that both temperature and food level seemed to modulate the relative expression of all these 11 genes potentially involved in the biomineralization process (Figure 3). Whatever the genes and environmental conditions tested, similar results were observed in the relative abundance of transcripts in the two pooled RNA mantle samples, each of which originated from five distinct animals (Figure 3).

For three (*Pif 177, MSI60, and shematrin 9*) out of the 11 genes tested, food level was found to change significantly (SHR non parametric ANOVA, p<0.05, table 2) the relative transcript abundance, whatever the temperature tested. High food level significantly increased expression of the genes *Pif 177* (Figure 3G) and *shematrin 9* (Figure 3C), while significantly decreased expression of the gene MSI60 (Figure 3F).

**Table 2:** Significance level of Scheirer-Ray–Hare (SHR) non-parametric ANOVA of calcifying genes expression level according to food and temperature levels.

Factor	df	PIF 177	MSI60	Pearlin	Linkine	Nacrein	Shem8	Shem9	MNP88	MP10	KRMP7	Fibronectin
Food	1	**0.02**	**0.02**	NS	NS	NS	NS	**0.00**	NS	NS	NS	NS
Temperature	2	NS	NS	**0.01**	**0.01**	**0.03**	**0.02**	NS	**0.02**	**0.02**	NS	**0.05**
Food/Temperature	2	NS	NS	NS	NS	NS	NS	NS	NS	NS	NS	NS

Among the 11 tested genes, 7 of them (*mantle protein 10*, *MPN88, fibronectin 1, shematrin 8, pearlin, linkine and nacrein)* were significantly regulated by temperature (SHR non parametric ANOVA, p<0.05, table 2). Three different expression patterns were shown according to the pairwise comparison of temperature using the Mann-Witney test: 1) a gradual down-regulation of gene expression for *fibronectin1* (Figure 3E); 2) up-regulation of gene expression at low temperature (21°C) only, for *mantle protein 10* (Figure 3B), *MPN88* (Figure 3D), *shematrin 8* (Figure 3C) down-regulation of gene expression at medium temperature (25°C) only, for *pearlin* (Figure 3H), *linkine* (Figure 3J) *and nacrein* (Figure 3K).

Lastly, expression of *KRMP7* (Figure 3A) gene coding for a prismatic protein did not change significantly according to food and temperature level (SHR non parametric ANOVA, p<0.05, table 2).

### Shell deposit rate is positively correlated with mantle gene expression of *Pif 177* and *shematrin 9*

Spearman’s correlation coefficient *rho* was calculated in order to measure the strength of the relationship of *P. margaritifera* mantle gene expression with SDR. Among the 11 studied genes, three showed relative expression correlated with SDR. *Pif 177* and *shematrin 9* were positively correlated with SDR, meaning that shell growth was higher when the relative expression of these genes increased. *MSI60* was negatively correlated to SDR (table 3). The global control pattern of the 11 studied genes and their involvement in shell growth is summarized in table 4.

**Table 3:** Spearman’s coefficient correlation rho between relative matrix gene expression and SDR.

Gene	SDR
PIF 177	**0.569**
MSI60	**–0.514**
Pearlin	–0.150
Linkine	0.165
Nacrein	0.178
Shematrin 8	0.142
Shematrin 9	**0.763**
MPN88	0.188
Mantle protein 10	0.187
KRMP7	0.250
Fibronectin1	0.202

Correlations were significant (in bold) at the 0.05 level when r<0.28 or >–0.28 (n = 49).

**Table 4:** Regulation pattern of genes according to temperature (T), food (F) and shell deposition rate (SDR).

Genes	Shell layer	Regulation pattern(T/F/SDR)
PIF 177	nacre	0/+/+
MSI60	nacre	0/−/−
Pearlin	nacre	−/0/0
Linkine	nacre	−/0/0
Nacrein	prism and nacre	−/0/0
Shematrin 8	prism and nacre	−/0/0
Shematrin 9	prism	0/+/+
MPN88	prism	−/0/0
Mantle protein 10	prism	−/0/0
KRMP7	prism	0/0/0
Fibronectin1	prism	−/0/0

## Discussion

Bivalve growth is known to be strongly influenced by environmental conditions such as food supply and water temperature [28]–[30]. The aim of this study was to simultaneously evaluate *P. margaritifera* shell growth and the expression level of genes encoding proteins of the shell matrix in the mantle depending on environmental conditions (microalgal concentration and temperature). We showed that both food level and temperature are correlated with shell deposition rate at the ventral side of the shell, but also with the level of expression of genes coding for proteins involved in this biomineralization process.

### Temperature and food influence shell growth in *Pinctada margaritifera*

The present study provides new information on the shell growth adaptive responses of the pearl oyster *P. margaritifera* to different temperature and food parameters. In bivalves, the literature provides little information about the environmental determinism of shell growth. Temperature influences shell microstructure of the bloody clam *Scarphaca broughtonii* [31]. The thickness of its shell microstructures is synchronized with seasonal temperature changes and the lamellar structure thickens during high temperatures in summer [31]. Contrastingly, below 20°C, the shell growth pattern of the mollusc bivalve *Ruditapes philippinarum* is not correlated with temperature [32]. In the pearl oyster *P. margaritifera,* [10] revealed a negative impact of temperature on the shell growth above 30°C. These thermal thresholds are probably specific to the ecological limits of these species. Ivanina et al. [33] underlined that elevated temperature negatively affect bioenergetics of some bivalves that impact physiological processus. In the present study, we showed that the shell deposit rate is temperature-dependant and it increases by a factor of three when the temperature increases from 21 to 28°C, that is the normal range of temperature in tropical area such as French Polynesia. Previous study, on the shell formation of *P. margaritifera* in Takapoto lagoon (French Polynesia) where the annual mean temperature is of 28.2°C (in the range of 26.5°C to 29.2°C), estimated that the nacreous deposition rate was in the range of 2 to 7 µm.d^−1^ [9]. These data are in accordance with our findings, where we showed that the range was between 1 to 9 µm.d^−1^ at 25°C and 2 to 12 µm.d^−1^ at 28°C.

Recently, Linard et al. [12] showed that microalgal concentration had a strong influence on nacre deposition rate in *P. margaritifera* and that this process induced a modification of the thickness of nacre tablets of the shell. The present study shows results on the influence of the availability of food on the shell deposit rate which was multiplied by almost 6 times between the 2 food levels tested. Food governs the processes of energy management, from the acquisition to the allocation. Chavez et al. [34] evidences that a high food level induces a high scope for growth and energy storage in *P. margaritifera*. The energy allocated to the shell growth can reach half of the total energy available for growth [35].

### Temperature and food modulate mantle gene expression

Results showed that a high concentration of microalgae significantly changes the abundance of transcripts of 3 of the 11 studied genes encoding proteins of the shell matrix. The over-abundance of *shematrin 9* and *Pif 177* transcripts in high-fed pearl oysters could result from a higher energy transfer to mantle cells implied in matrix protein secretion for shell building. Inversely *MSI60*, a major gene involved in the formation of nacreous aragonites [36]–[37], [17], [19], seemed down regulated by food. The low transcript abundance of *MSI60* probably resulted from a different mechanism of gene regulation. This result on the *MSI60* gene regulation by food is interesting and may be correlated to the data of Inoue et al. [38]. They have shown that the expression variation of *MSI60* gene, in recipient pearl oysters, influences the mineralization and the pearl quality.

The global control pattern of gene expression by temperature is a down-regulation. After 2 months at 28°C, we observed a decrease in the expression level of 8 of the 11 monitored genes coding for proteins of the shell matrix. In contrast, *MSI60*, *linkine* and *shematrin 9* genes are not regulated by temperature. These results could be linked to those of Pouvreau and Prasil [10] that revealed a negative impact of high temperature (30°C) on *P. margaritifera* shell growth. These thermal thresholds are probably specific to the ecological limits of the pearl oyster *P. margaritifera*. There are few data on the impact of temperature on mantle gene expression in bivalves in the literature. Liu et al. [39] observed no effect of temperature between 27 and 30°C on the expression of five mantle genes (*calmodulin, aspein, nacrein, shematrin 7* and *hsp70*) in the pearl oyster *Pinctada fucata*. Hence, our study is the first to find a correlation between the level of expression of genes involved in the biomineralization process and environmental conditions. However, it remains difficult to pinpoint the exact impact of environmental conditions on molecular and physiological mechanisms underlying the biomineralization process of the shell, since many steps of this process are still uncharacterized. Transcripts of the studied genes are expressed in the calcifying mantle and the proteins identified in the mineralized structure. However, the molecular regulatory mechanisms upstream from the secretory cascade remain unknown [15], [23], [40].

### Molecular control of shell growth by environmental factors

As environmental factors regulate mantle genes expression of genes, it was interesting to correlate the shell matrix protein gene expression level with the shell growth rate. The correlation analysis showed that only two genes (*shematrin 9* and *Pif 177)* were related to shell growth rate. The transcript levels of these two genes were also controlled by food level. These results suggest that additional energy provided by food may enhance shell deposit rate by involving the shell matrix proteins *Pif 177* and *shematrin 9*. In the pearl producing bivalves *P. fucata* and *P. margaritifera*, *shematrin 9* transcripts are expressed in the mantle edge, which indicates that this protein family is expressed as components of the prism matrix [15], [41]. Results from Jackson *et al.* [42] on *P. maxima* also suggest that proteins from the shematrin family might be located in the inner nacre part of the shell. Measurement of the expression level of the *shematrin 9* gene in the mantle therefore appears to be an indicator of interest for the evaluation of the biomineralization activity of the *P. margaritifera* shell under varying environmental conditions.

For the cultured pearl oyster industry and from an applied point of view, the level expression of those two shell growth genes, *Pif 177* and s*hematrin 9*, constituted also interesting molecular tool for genetic selection of *P. margaritifera* with high potential for nacre deposition rate [43]. In fact, significant variation of nacre thickness and nacre weight were observed between wild donor oysters [44] and between farmed donor families [45]. Overall, the use of a multi-trait approaches, which take into account studies of environmental effects, genes expression and quantitative genetic control may be an effective strategy to improve pearl quality.

## Conclusion

In this study, we showed that environmental conditions, such as food and temperature, directly influence *P. margaritifera* shell growth and modulate gene expression level of shell matrix proteins in the mantle, the mineralizing tissue of the shell. These results establish a basis for the environmental control of shell growth at the molecular level. This study also provides preliminary information on the adaptive responses of the pearl oyster *P. margaritifera to* environmental conditions, such as global climate change and its consequences.
